# Timed flat infusion of 5-fluorouracil increases the tolerability of 5-fluorouracil/docetaxel regimen in metastatic breast cancer: a dose-finding study

**DOI:** 10.1038/sj.bjc.6601971

**Published:** 2004-08-03

**Authors:** C Ficorella, M F Morelli, E Ricevuto, K Cannita, G Porzio, P Lanfiuti Baldi, G Cianci, ZC Di Rocco, C Natoli, N Tinari, F De Galitiis, F Calista, P Marchetti

**Affiliations:** 1 Medical Oncology, University of L'Aquila, Italy; 2 Department of Oncology and Neuroscience Section of Medical Oncology, University of Chieti, Italy

**Keywords:** breast cancer, docetaxel, 5-fluorouracil

## Abstract

A dose-finding study was undertaken to determine the maximum-tolerated dose, and the recommended dose of docetaxel in combination with 12-h timed (22:00–10:00) flat infusion of 5-fluorouracil (5-FU) in metastatic breast cancer patients. This schedule seems to reduce the occurrence of stomatitis of the docetaxel and infusional 5-FU regimen.

Biological circadian rhythms may affect the tolerability and efficacy of 5-fluorouracil (5-FU). The chrono-modulated regimen of 5-FU, with maximum delivery at 1600, reduces 5-FU toxicity and increase 5-FU median dose intensity (Lévi, 1997). We associated 12-h timed (22:00–10:00) flat infusion (^22:00–10:00^TFI) of 5-FU to docetaxel (dTX) to exploit the increased activity of dehydropyrimidine dihydrogenase in human mononuclear cells and the reduced cell replication activity of human bone marrow and of the oral and rectal mucosa, during the night hours compared to daytime (Caussanel *et al*, 1990; Smaaland *et al*, 1991). ^22:00–10:00^TFI traces the 12 h circadian-timed infusion of 5-FU (22:00–10:00 with maximum delivery at 0400) and may contribute to increasing its tolerability, making administration easier than the chrono-modulated infusion. The objectives of the present dose-finding study were to determine the maximum-tolerated dose (MTD) and the recommended dose (RD) of this 5-FU/dTX schedule in metastatic breast cancer patients.

## PATIENTS AND METHODS

Patient eligibility criteria were: histologically or cytologically documented breast cancer and proven metastatic or recurrent breast cancer; measurable or evaluable metastatic disease; WBC count ⩾4 × 10^3^ mm^−3^, neutrophils ⩾2 × 10^3^ mm^−3^, platelets ⩾100 × 10^3^ mm^−3^, hemoglobin ⩾10 g dl^−1^, serum creatinine ⩽1. 2 mg dl^−1^, total bilirubin ⩽1.5 times the upper normal limit, AST and ALT ⩽1.5 times the upper normal limit; age between 18 and 75 years; World Health Organisation (WHO) performance status ⩽2. The study was approved by the Local Ethics Committee. Prior chemotherapy for metastatic disease was not allowed. The exclusion criteria included: peripheral neuropathy, uncontrolled infection, diabetes and cardiac disease.

The treatment schedule consisted of a 1 h i.v. dTX (Taxotere®) infusion on day 1 and a 12-h timed (22:00–10:00) flat i.v. 5-FU (Fluorouracil TEVA®) infusion, over 5 days, every 21 days. Four escalation dose levels of dTX plus 5-FU were planned: 5-FU 700 mg m^−2^ day^−1^ associated to dTX 80 and 85 mg m^−2^ in the first two dose levels, respectively; then, 5-FU dose levels were increased to 800 and 900 mg m^−2^ day^−1^ in the other two steps. Implantation of a venous access device was required for 5-FU administration via a programmed portable pump (Cadd-Plus, Sevit) that administered 5-FU at a given constant rate for a period of 12 h. Treatment was routinely administered on an outpatient basis. The planned dose-escalation strategy combined the intra- and interpatient approach (Simon *et al*, 1997). The MTD was defined as the dose at which at least 50% of patients developed dose-limiting toxicity (DLT). In case of DLT, as defined in the treatment plan, treatment was continued at the dose level immediately below. This dose was the RD for phase II trials. Granulocyte colony-stimulating factor (G-CSF) was administered at the first occurrence of grade (G)4 neutropenia due to its high expected rate, in order to prevent febrile neutropenia and treatment delays; in the subsequent cycles, prophylactic G-CSF treatment was administered for 5 days, after the occurrence of G4 neutropenia. Complete blood cell count was performed on days 1, 6, 8, 12, every cycle.

Dose-limiting toxicity included: febrile neutropenia requiring i.v. antibiotics or G4 neutropenia resistant to G-CSF administration (failure to recover neutrophils ⩾1.5 × 10^3^ mm^−3^ on day 21 of each cycle); grade 4 thrombocytopenia; hemoglobin <6.5 g dl^−1^; grade 3–4 nonhaematological toxicity (excluding alopecia and nausea). Toxicity was graded according to the National Cancer Institute Common Toxicity Criteria (NCI-CTC). Before entering the study, each patient underwent medical history, physical examination, complete blood cell count, serum biochemistry, computed tomography of the chest and abdomen, bone scan, an electrocardiogram and other investigations as clinically indicated. Tumour imaging was repeated every three treatment cycles. Tumour response was assessed according to WHO response criteria. Time to disease progression and survival were assessed using the methods of Kaplan and Meier.

## RESULTS

Fourteen patients were enrolled. A summary of baseline patient characteristics is illustrated in Table 1

**Table 1:** Patient characteristics

	**No. (%)**
Number of patients	14 (100)
Median age (years)	52
Range	36–69
*WHO*^a^ *performance status*	
0	11 (78)
⩾1	3 (22)
*Adjuvant therapy*	
Chemotherapy with anthracyclines	10 (71)
Chemotherapy without anthracyclines	3 (21)
Hormonal therapy	10 (71)
*Previous metastatic breast cancer therapy*	
Hormonal therapy	1 (7)
*Disease sites*	
Soft tissue and skin	2 (14)
Liver	6 (43)
Lung and pleura	7 (50)
Bone	4 (29)
Brain	1 (7)
*Number of organs involved*	
1	6 (43)
2	8 (57)

a WHO=World Health Organisation.

. A total of 88 cycles were administered, with a median of six cycles per patient (1–12); the median number of dose-escalations per patient was 2 (1–4).

The fourth dose level (dTX 85 mg m^−2^ and 5-FU 900 mg m^−2^ day^−1^) represented the MTD (Table 2

**Table 2:** DLTs according to the dose-escalation scheme

**Dose levels**	**Docetaxel (mg m^−2^)–5-FU (mg m−^2^ day^−1^)**	**No. of patients^a^**	**No. of cycles**	**No. of patients with DLT/total patients (%)**	**No. of cycles with DLT/total cycles (%)**	**DLTs**
I	80–700	5	15	—	—	—
II	85–700	8	12	—	—	—
III	85–800	12	34	—	—	—
IV	85–900	10	27	5/10 (50)	5/27 (18)	Two G3 diarrhoea
						Two G4 diarrhoea
						One G3 mucositis and febrile neutropenia

a Intra- and interpatient dose escalation.DLT=dose-limiting toxicity; 5-FU=5-fluorouracil.

): ten patients were treated and 27 cycles were administered. Dose-limiting toxicities were observed in five of 10 patients (50%): two patients experienced grade (G)4 diarrhoea; two patients, G3 diarrhoea; one patient, G3 mucositis (10%) and febrile G3 neutropenia. Each of the five patients received subsequent cycles at the third dose level without showing DLTs.

The third dose level (dTX 85 mg m^−2^ and 5-FU 800 mg m^−2^ day^−1^) represented the RD: 12 patients were treated and 34 cycles were administered. Dose-limiting toxicities were not observed and chemotherapy was well tolerated (Table 3

**Table 3:** Nonhaematological toxicity at recommended dose and MTD

	**Cycles**				**Patients^a^**			
**Dose levels**	**III**		**IV**		**III**		**IV**	
**Docetaxel (mg m^−2^)-5-fluorouracile (mg m^−2^ day^−1^)**	**85–800**		**85–900**		**85–800**		**85–900**	
**Number**	**34**		**27**		**12**		**10**	
**Grade**	**(1–2)**	**(3–4)**	**(1–2)**	**(3–4)**	**(1–2)**	**(3–4)**	**(1–2)**	**(3–4)**
Nausea	10	—	6	—	5	—	3	—
(%)	(29)		(22)		(42)		(30)	
Vomiting	1	—	5	2	1	—	1	1
(%)	(3)		(18)	(7)	(8)		(10)	(10)
Diarrhoea	7	—	6	4	5	—	2	4
(%)	(21)		(22)	(15)	(42)		(20)	(40)
Stomatitis	18	—	10	1	4	—	5	1
(%)	(53)		(37)	(4)	(33)		(50)	(10)
Neurosensory	3	—	11	—	1	—	1	—
(%)	(9)		(41)		(8)		(10)	
Dermatitis	—	—	6	—	—	—	2	—
(%)			(22)				(20)	
Asthenia	8	—	16	—	4	—	6	—
(%)	(23)		(59)		(33)		(60)	
Fluid retention	6	—	15	—	3	—	3	—
(%)	(18)		(55)		(25)		(30)	
Nail toxicity	4	—	10	—	2	—	2	—
(%)	(12)		(37)		(17)		(20)	

a Intra- and interpatient dose escalation.MTD=maximum-tolerated dose.

): Grade 1–2 diarrhoea in 42% of patients and 21% of cycles; G1–2 stomatitis in 33% of patients and 53% of cycles; G 1–2 nausea/vomiting was observed in 50% of patients and 32% of cycles. Grade 2 alopecia was present in all patients. No toxic death was observed. One case of thrombosis related to the venous access device was observed.

At the RD, G4 neutropenia was observed in 50% of patients (six of 12) and in 47% of cycles (16 of 34); G3–4 neutropenia 79% (27 of 34). Overall, it has been observed in 36% (32 of 88) of cycles. Patients showing G4 neutropenia were all treated with G-CSF. The median time to neutrophil nadir was on day 8 and the median duration of G-CSF administration was 5 days. One patient experienced febrile G3 neutropenia at MTD.

The median dose intensity administered was 1333 mg m^−2^ week^−1^ (900–1500) and 28.3 mg m^−2^ week^−1^ (17–28.3) for 5-FU and dTX, respectively. Patients received an average dose intensity of 1321 mg m^−2^ week^−1^ (*α*: 0.05, CI: ±49) for 5-FU and of 27.7 mg m^−2^ week^−1^ (*α*: 0.05, CI: ±0.3) for dTX.

In all, 13 patients were assessable for treatment efficacy. Two complete and six partial responses were observed giving an overall response rate of 61% (*α*: 0.05, CI: ±28). The median time to progression was 10 months (4–28 months) and the median overall survival was 25 months (4 to 46+ months).

## DISCUSSION

The RD for phase II studies of this dTX/5-FU schedule (dTX 85 mg m^−2^, day 1; 5-FU 800 mg m^−2^ day^−1^ in ^22:00–10:00^TFI) shows a projected dose intensity equivalent to that proposed by Lortholary *et al* (2000) (dTX 85 mg m^−2^ day 1; 5-FU 750 mg m^−2^ day^−1^ 5-day continuous infusion, every 3 weeks) (). These RDs were well tolerated with the use of G-CSF. No DLTs were observed in the 12 treated patients and 34 administered cycles. Nonhaematological toxicity was characterised by G1–2 diarrhoea and stomatitis. The earlier dose-finding studies of docetaxel in combination with 5-FU in advanced breast cancer show that mucositis (stomatitis and diarrhoea) and neutropenia represented the DLTs at the MTD (Ando *et al*, 1998; Petit *et al*, 1999). In our study and in that by Lortholary *et al*, neutropenia was common but recovered after medical G-CSF management.

In the study by Lortholary *et al*, few instances of febrile neutropenia were observed and stomatitis represented the DLT at the MTD; at the RD, 40% (two of five) of patients experienced G3 dose-limiting stomatitis and G4 neutropenia was reported in four of five patients (Lortholary *et al*, 2000). The recently published phase II study by Lortholary confirms that: neutropenia is the most common toxic event (G3–4, 54% of patients; febrile neutropenia, 24% of patients); stomatitis G3–4 is a frequent event (26% of patients, approximately 6% of cycles); G3 diarrhoea occurs in 7% of patients (1% cycles) (Lortholary *et al*, 2003). Even if in the present study the use of G-SCF did not allow to report the duration of neutropenia, no instance of febrile neutropenia was observed at the RD.

In the present study, at the RD, nonhaematological toxicity was characterised by G1–2 diarrhoea (42% of patients and 21% of the cycles) and G1–2 stomatitis (33% of the patients and 53% of the cycles). The present data suggest that the ^22:00–10:00^TFI of 5-FU may increase the nonhaematological tolerability of the 5-FU/dTX regimen, particularly by reducing the occurrence of dose-limiting stomatitis.

Among the oral fluoropirimidine formulations, capecitabine mimicks protracted infusion of 5-FU. The present data are particularly significant to this concern in view of a potential comparison between the most tolerable and effective schedules of dTX and 5-FU or capecitabine in metastatic breast cancer.

## References

[bib1] Ando M, Watanabe T, Sasaki Y, Ying DF, Omuro Y, Katsumata N, Narabayashi M, Tokue Y, Fujii H, Igarashi T, Wakita H, Ohtsu T, Itoh K, Adachi I, Taguchi T (1998) A phase I/II study of docetaxel in combination with 5-fluorouracil for advanced or recurrent breast cancer. Br J Cancer 77: 1937–1943 9667671 10.1038/bjc.1998.321 PMC2150334

[bib2] Caussanel JP, Levi F, Brienza S, Misset JL, Itzhaki M, Adam R, Milano G, Hecquet B, Mathe G (1990) Phase I trial of 5-day continuous venous infusion at circadian rhythm modulated rate compared with constant rate. J Natl Cancer Inst 82: 705–714 10.1093/jnci/82.12.1046 2348469

[bib3] Lévi F (1997) Chronopharmacology of anticancer gents. In Handbook of Exeperimental Pharmacology: Physiology and Pharmacology of Biological Rhythms-cancer Chemotherapy, Redfern PH, Lemmer B (eds) pp 299–301, Berlin: Springer-Verlag

[bib5] Lortholary A, Delozier T, Monnier A, Bourgeois H, Bougnoux P, Tubiana-Mathieu N, Riffaud JCh, Besson D, Lotz V, Gamelin E (2003) Phase II multicentre study of docetaxel plus 5-fluorouracil in patietnts with anthracycline-pretreated metastatic breast cancer. Br J Cancer 88: 1669–1674 12771978 10.1038/sj.bjc.6600989 PMC2377146

[bib4] Lortholary A, Maillard P, Delva R, Boisdron-Celle M, Perard D, Vernillet L, Besenval M, Gamelin E (2000) Docetaxel in combination with 5-fluorouracil in patients with metastatic breast cancer previously treated with anthracycline-based chemotherapy: a phase I, dose-finding study. Eur J Cancer 36: 1773–1780 10974625 10.1016/s0959-8049(00)00176-3

[bib6] Petit T, Aylesworth C, Burris H, Ravdin P, Rodriguez G, Smith L, Peacock N, Smetzer L, Bellet R, Von Hoff DD, Rowinsky EK (1999) A phase I study of docetaxel and 5-fluorouracil in patient with advanced solid malignancies. Ann Oncol 10: 223–229 10093693 10.1023/a:1008356025108

[bib7] Simon R, Freidlin B, Rubinstein L, Arbuck SG, Collins J, Christian MC (1997) Accelerated titration designs for phase I clinical trials in oncology. J Natl Cancer Inst 89: 1138–1147 9262252 10.1093/jnci/89.15.1138

[bib8] Smaaland R, Laerum OD, Lote K, Sletvold O, Sothern RB, Bjerknes R (1991) DNA synthesis in the human bone marrow is circadian stage dependent. Blood 77: 2603–2611 2043764

